# Neuroimaging basis in the conversion of aMCI patients with APOE-ε4 to AD: study protocol of a prospective diagnostic trial

**DOI:** 10.1186/s12883-016-0587-2

**Published:** 2016-05-12

**Authors:** Guan-Qun Chen, Can Sheng, Yu-Xia Li, Yang Yu, Xiao-Ni Wang, Yu Sun, Hong-Yan Li, Xuan-Yu Li, Yun-Yan Xie, Ying Han

**Affiliations:** Department of Neurology, XuanWu Hospital of Capital Medical University, Beijing, 100053 China; Center of Alzheimer’s Disease, Beijing Institute for Brain Disorders, Beijing, 100053 China; Department of Neurology, Tangshan Gongren Hospital, Tangshan, 063000 China; Department of Neurology, Civil Aviation General Hospital, Beijing, 100123 China

**Keywords:** APOE4 allele, Alzheimer’s disease, Amnestic mild cognitive impairment, Neuroimaging techniques, Multimodality, Longitudinal study

## Abstract

**Background:**

The ε4 allele of the Apolipoprotein E gene (APOE-ε4) is a potent genetic risk factor for sporadic Alzheimer’s disease (AD). Amnestic mild cognitive impairment (aMCI) is an intermediate state between normal cognitive aging and dementia, which is easy to convert to AD dementia. It is an urgent problem in the field of cognitive neuroscience to reveal the conversion of aMCI-ε4 to AD. Based on our preliminary work, we will study the neuroimaging features in the special group of aMCI-ε4 with multi-modality magnetic resonance imaging (structural MRI, resting state-fMRI and diffusion tensor imaging) longitudinally.

**Methods/Design:**

In this study, 200 right-handed subjects who are diagnosed as aMCI with APOE-ε4 will be recruited at the memory clinic of the Neurology Department, XuanWu Hospital, Capital Medical University, Beijing, China. All subjects will undergo the neuroimaging and neuropsychological evaluation at a 1 year-interval for 3 years. The primary outcome measures are 1) Microstructural alterations revealed with multimodal MRI scans including structure MRI (sMRI), resting state functional MRI (rs-fMRI), diffusion tensor imaging (DTI); 2) neuropsychological evaluation, including the World Health Organization-University of California-LosAngeles Auditory Verbal Learning Test (WHO-UCLA AVLT), Addenbrook’s cognitive examination-revised (ACE-R), mini-mental state examination (MMSE), Montreal Cognitive Assessment (MoCA), Clinical Dementia Rating scale (CDR).

**Discussion:**

This study is to find out the neuroimaging biomarker and the changing laws of the marker during the progress of aMCI-ε4 to AD, and the final purpose is to provide scientific evidence for new prevention, diagnosis and treatment of AD.

**Trial Registration:**

This study has been registered to ClinicalTrials.gov (NCT02225964, https://www.clinicaltrials.gov/) in August 24, 2014.

## Background

Alzheimer’s disease (AD) is a common neurodegenerative disorder, which is increasingly prevalent among the elderly. AD patients usually complain of memory impairment and serious decline of the daily living abilities. The aMCI is an intermediate state between normal cognitive aging and dementia, which is easily progressed to AD dementia. Thus, the effective therapy of AD is to be delivered once the diagnosis is made at the very early stages of AD, ideally in the phase of aMCI. The human apolipoprotein E (APOE) gene has three polymorphic alleles (E2, E3, and E4) [1]. APOE-ε4 was confirmed to be related with sporadic AD risks by increasing the risk of developing AD and reducing the age of onset [2]. Therefore, it is an urgent requirement in the field of cognitive neuroscience to reveal the underlying neurological mechanism of the conversion of aMCI to AD in the patients with APOE-ε4.

Although the cognition evaluation is simple, the accuracy of the diagnosis depends on the professional skills of the doctor and the collaboration of patients. On the other hand, MRI might be a promising strategy for early diagnosis of AD [3]. MRI might be the best diagnosis method for AD by mapping both structures and functions of the human brain. Structurally, studies showed that brain atrophy in AD patients first appears in the medial temporal lobe (MTL), and then involves the parietal lobe, frontal lobe and occipital lobe, and finally the anterior cingulate cortex [4, 5]. Consistent with these findings, evidence from our team has demonstrated the progressive alterations of brain structures during the progression to AD [6]. Compared with APOE-ε4 non-carriers, the carriers exhibited greater medial temporal lobe atrophy [7]. APOE-ε4 is an independent risk factor for the hypotrophy hippocampus in patients with AD and MCI [8]. Decreased gray matter volume mainly restricts in the right brain area for aMCI-ε4 patients [9]. Not the degree of atrophy, but the rate of atrophy in a certain region of brain demonstrate close relationship with the AD progression [10]. The rate of hippocampal atrophy is suggested to be an early marker of incipient memory decline and dementia [11]. The longitudinal study indicated that rate of volumetric loss was significantly greater among MCI-ε4 compared with non-carriers [12]. Compared with structural MRI, diffusion tensor imaging (DTI) is a more sensitive method for detecting white matter damage [13]. A short-term follow-up study showed that AD patients had lower fractional anisotropy (FA) than controls in the fornix and anterior portion of the cingulum bundle, and these FA values were positively correlated with cognitive score [14]. The APOE-ɛ4 modulates white matter (WM) before clinical manifestations and cognitive impairment [15]. The changes of white matter is accompanied with the cognitive function decline in APOE-ε4 carriers [16]. Previous studies suggested that rs-fMRI is a potential functional biomarker for cognitive impairment. The aMCI subjects showed that posterior cingulate cortex/precuneus (PCC/PCu) hyper-functional connectivity was found at baseline, while a substantial decrease of these connections was observed at follow-up [17]. Our team observed that the aMCI patients had decreased amplitude of low-frequency fluctuations (ALFF) values in the PCC/PCu, anterior medial prefrontal cortex (aMPFC), hippocampus/parahippocampal gyrus (PHG), basal ganglia, and prefrontal regions, and increased ALFF values mainly in several occipital and temporal regions [18]. APOE-ε4 exerts an influence on brain function in patients with MCI. Using whole-brain pulsed arterial spin labeling (ASL) magnetic resonance imaging suggest that cognitive status and APOE genotype have interactive effects on cerebral blood flow (CBF) [19]. MCI-ε4 patients demonstrated significantly increased CBF [20]. However, deficits exist in these studies that using rs-fMRI to reveal the effect of APOE-ε4 on MCI patients.

For the purpose of early diagnosis, the researchers have been focusing on the aMCI window. Although previous studies provide important information for the conversion of aMCI to AD, they were based on the single modality neuroimaging analysis. Moreover, previous studies ignored one of the major factors that affects the course of the MCI converting to AD is the APOE-ε4. Thus, the purpose of the current trial is to longitudinally investigate the multi-modal (structural MRI, resting state-fMRI and diffusion tensor imaging) neuroimaging characteristics of aMCI-ε4 patients. We hope to provide scientific evidence for more effective prevention, diagnosis and treatment of AD.

## Methods/Design

**Fig 1:**
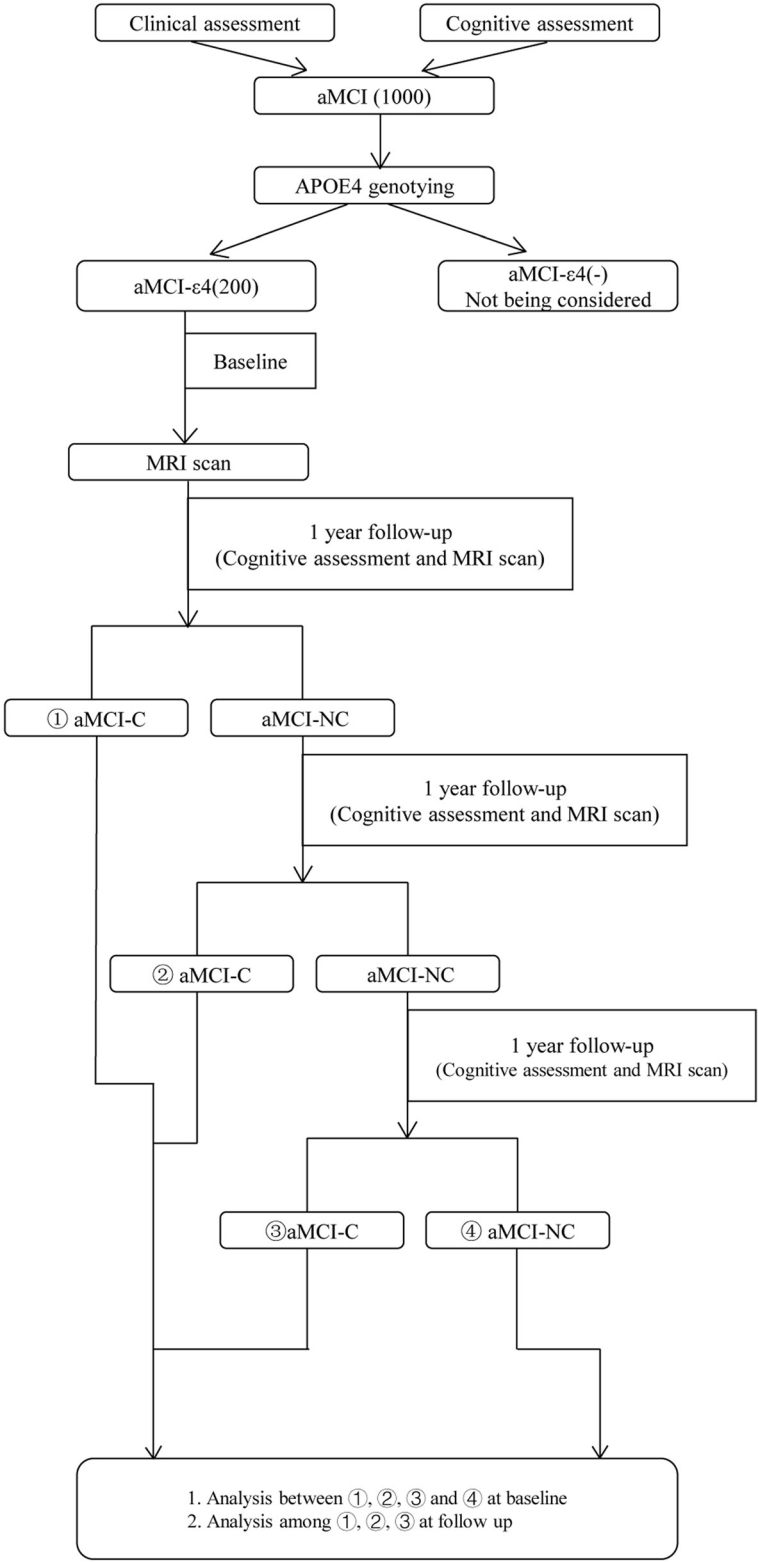
Flowchart of the current prospective diagnostic trial

The scheme of the current prospective trial is described in Fig. 1.

### Subject

1. Subjects inclusion: subjects with aMCI included the following: (1) memory complaint, preferably confirmed by an informant; (2) a single domain or multi domain cognitive decline; abnormal objective memory impairment documented by the scores falling 1.5 SD below the age and education matched-specific norms on memory test; being free from dementia according to the Diagnostic and Statistical Manual of Mental Disorders, Fourth Edition, revised (DSM-IV-R); (3) objective memory impairment, cutoff points of MiniMental State Examination (MMSE) score: 19 (no formal education), 22 (1 to 6 years of education), and 26 (7 or more years of education); cutoff points of Montreal Cognitive Assessment (MoCA): 13 (no formal education), 19 (1 to 6 years of education), and 24 (7 or more years of education) [21]; a Clinical Dementia Rating (CDR) score of 0.5; (4) the Han nationality, right-handed (the Edinburgh handedness scale score >40 points); no fixed dentures and can accept MRI scan; (5) able to complete 3 years follow-up.
2. Subjects exclusion: (1) those who have a clear history of stroke; (2) severe depression that led to mild cognitive impairment (Hamilton Depression Rating Scale score ≥ 24 points); (3) other nervous system diseases, which can cause cognitive impairment (such as brain tumors, Parkinson's disease, encephalitis, and epilepsy); (4) cognitive impairment caused by traumatic brain injury; (5) other systemic diseases, which can cause cognitive impairment, such as thyroid dysfunction, severe anemia, syphilis, and HIV; (6) a history of psychosis or congenital mental growth retardation; and (7) subjects with contraindications for MRI.

### Neuropsychological assessments

All subjects will receive a standardized clinical and neuropsychological evaluation, including WHO-UCLA AVLT, ACE-R, MMSE, MoCA, CDR.

### APOE genotype

After the neuropsychological assessments and before the MRI scan, genomic DNA will be extracted from peripheral blood using a Blood Genomic DNA Extraction Kit. The APOE genotypes will be determined by a restriction enzyme polymerase chain reaction technique. Only subjects carrying the gene of E3/E4 or E4/E4 will be enrolled in this study group.

### Magnetic resonance brain imaging

#### Imaging protocol

Imaging data will be acquired using a 3.0 T Trio Siemens scanner at XuanWu Hospital, Capital Medical University. For each participant, conventional brain T1-weighted (T1WI), T2-weighted (T2WI) and fluid-attenuated inversion recovery (FLAIR) images will be obtained to exclude serious brain diseases. Brain MR images will be inspected by an experienced neuroradiologist (with over 5-year experience).

sMRI. using a sagittal MP-RAGE sequence with the following imaging parameters: TR = 1900 ms; TE = 2.2 ms; inversion time = 900 ms; flip angle = 9^。^; FOV = 256 mm × 256 mm; matrix = 256 × 256;176 slices, thickness = 1.0 mm.

rs-fMRI. using an echo-planar imaging sequence with the following parameters: repetition time (TR) = 2000 ms, echo time (TE) = 40 ms, flip angle (FA) = 90∘, number of slices = 28, slice thickness = 4 mm, gap = 1 mm, voxel size = 4 × 4 × 4 mm^3^, and matrix = 64 × 64. Participants will be asked to lie quietly in the scanner with their eyes closed during data acquisition. Each scan lasted for 478 s.

DTI. using an echo planar imaging (EPI) sequence in 32 independent, non-collinear directions of a b-value = 1000 s/mm^2^, and one additional image with no diffusion weighting (b = 0). TR = 11000 ms, TE = 98 ms, flip angle = 90^。^, field of view (FOV) = 256 mm × 256 mm, imaging matrix = 128 × 128, number of slices = 60, and slice thickness = 2 mm. Three acquisitions will be averaged to increase the signal-to-noise ratio.

#### MRI image analysis

sMRI data analysis. Voxel-based morphometry (VBM) with DARTEL will be used to characterize gray matter volume (GMV). Voxel-based morphometry with DARTEL will be performed using SPM8 (Welcome Trust Center for Neuroimaging, London, UK, http://www.filion.ucl.ac.uk/spm/software/spm8/). For the analysis of cortical thickness, we will use the software package of the Montreal Neurological Institute (MNI)(http://wiki.bic.mni.mcgill.ca/index.php/CIVET). This process includes structural image non-uniformity correction, spatial standardization, tissue segmentation (gray matter, white matter and cerebrospinal fluid), internal and external surface extraction of gray matter as well as the definition and measurement of cortical thickness.

rs-fMRI data analysis. Image preprocessing will be performed by using SPM8 (http://www.fil.ion.ucl.ac.uk/spm/) and Data Processing Assistant for Resting-State fMRI. The preprocessing procedures will be performed including removal of the first 10 volumes, slice timing, and head motion correction. All rs-fMRI data will be satisfied the criteria of spatial movement in any direction < 3 mm or 3^o^ and the subjects will be demonstrated no significant group differences in the head motion parameters (i.e, three translation and three rotation parameters). To normalize the fMRI data spatially, the T1-weighted images will be firstly registered to the mean functional data, and the resulting aligned T1 data set will be segmented and transformed into MNI space using the DARTEL toolbox, and a group template will be generated. Next, the motion-corrected functional volumes will be specially normalized to the group template using the transfer parameter estimated by DARTEL segmentation and resampled to 3 mm isotropic voxels. Further, the functional images will be spatially smoothed with a 4 mm Gaussian kernel. The linear detrend and temporal bandpass filtering (0.01 – 0.08 Hz) will be performed to reduce the influences of low-frequency drift and high-frequency physiological noise. Finally, several nuisance signals will be regressed out from the data, including the six motion parameters, the global, the white matter, and the cerebrospinal fluid signals.

Functional connectivity (FC) analysis using DPARSF software (http://www.restfmri.net/forum/DPARSF).

DTI data analysis. DTI data processing will be carried out using FSL software (FMRIB Software Library, http://www.fmrib.ox.ac.uk/fsl). Initially, eddy current correction will be run to correct gradient-coil distortions and small-head motions using affine registration to a reference image (b0 volume). The brain voxels of DTI data will be extracted using the Brain Extraction Tool (BET). The maps of diffusion tensor parameters including FA and MD will be calculated using DTI-FIT tool, which fits a diffusion tensor model to diffusion-weighted images for each voxel. Voxel-wise statistical analysis of the FA and MD data will be performed for regional differences using TBSS.

### Follow up

All aMCI-ε4 patients will undergo a follow-up review of approximately 3 years with 1-year interval. They will get the entire clinical examination, neuropsychological assessment and MRI scan during the baseline period. Individuals will come back to the XuanWu Hospital once 1 year for countercheck. All the evaluations are the same as that they receive during the first clinic visit. According to the diagnosis in the follow-up stage, aMCI-ε4 patients will be divided into converters and nonconverters to AD.

### Sample size calculation and statistics

A total of 1000 right-handed aMCI patients will be recruited in this study. aMCI-ε4 patients is about 26.6 %, and annual conversion rate of aMCI-ε4 patients convert is 15.9 % [22]. Thus, we plan to recruit 200 patients with aMCI-ε4. There will be about 90 aMCI-ε4 converted into AD after 3 years of follow-up.

Demographics and clinical characteristics of the subjects will be analyzed using SPSS 17.0. Two-sample two-tail *t* test or two-tail Pearson chi-square test will be used to explore differences of these data among two groups (converters and nonconverters). *P* < 0.05 is considered as with statistical significance.

For images analysis. To determine the difference between two groups (converters and nonconverters), we will perform a two-sample *t*-test. The significant level will be set at *P* < 0.05.

To determine the difference between intra-groups, we will perform a One-way analysis of variance (ANOVA). Further, Dunnett-*t* test will be performed for differences among intra-groups. The statistical threshold of the two test will both be set at *P* <0.05.

## Discussion

To our know, APOE-ε4 is a strong risk factor of AD. However, there has controversy about the effect of APOE-ε4 on the conversion of aMCI-ε4 to AD. A multivariate Cox regression model demonstrated that possession of an APOE-ε4 was the strongest predictor of clinical progression among aMCI [23]. Other studies of have found limited clinical applicability for prediction of outcome among aMCI [24, 25]. Moreover, Stern et al implied that APOE-ε4 is associated with a less aggressive form of AD [26]. Thus, previous studies have shown that simply depending on the psychometric tests will cause a noisy clinical data, and need biomarker to clarify the pathogenic mechanism of APOE-ε4, such as MRI, PET (positron emission tomography), and cerebrospinal fluid (CSF) examinations. Comparing to PET and CSF examinations, MRI ─ a noninvasive, nonradiation means ─ has been widely used with easy patient acceptance in both clinical and scientific studies.

Some limitations have to be took into account about this study. First, the trial's follow up time may be too short to be sured whether MCI-nonconverter would convert to AD in the future. Therefore, we will plan to continue to track the dynamic situations of these patients. Second, there has many technical problems of MRI. Data acquisition, data processing and data analysis of MRI are lack of standards. So far, no MRI model can be used for clinical diagnosis of AD. But we deeply believe that with the development of neuroimaging, diagnosis of AD with MRI will achieve better performance.

Here, this present study is to find out the neuroimaging biomarker and the changing laws of the marker during the progress of aMCI-ε4 to AD, and the final purpose is to provide scientific evidence for new prevention, diagnosis and treatment of AD.

## Ethics approval and consent to participate

Ethical approval of this has been obtained from the medical research ethics committee and institutional review board of XuanWu Hospital, Capital Medical University (approval number: [2014]011). All participation is based on written informed consent and the participants will be able to withdraw from the study at any time.

## Consent for publication

Written consent is obtained from each subject before publishing in this study.

## Availability of data and material

Not applicable.

## Abbreviations

AD: Alzheimer’s disease
ALFF: amplitude of low-frequency fluctuations
aMCI: amnestic mild cognitive impairment
aMPFC: anterior medial prefrontal cortex
ANOVA: one-way analysis of variance
APOE-ε4: ε4 allele of the Apolipoprotein E gene
ASL: arterial spin labeling
CDR: clinical dementia rating scale
CSF: cerebrospinal fluid
DPARSF: data processing assistant for resting-state fMRI
DTI: diffusion tensor imaging
FA: fractional anisotropy
FC: functional connectivity
FLAIR: fluid-attenuated inversion recovery
MD: mean diffusivity
MMSE: mini-mental state examination
MoCA: Montreal cognitive assessment
MRI: magnetic resonance imaging
MTL: medial temporal lobe
PCC: posterior cingulate cortex
PCu: precuneus
PET: positron emission tomography
PHG: parahippocampal gyrus
rs-fMRI: resting state functional magnetic resonance imaging
sMRI: structural magnetic resonance imaging
SPM8: statistical parametric mapping 8
T1WI: T1-weighted imaging
T2WI: T2-weighted imaging
TBSS: tract-based spatial statistics
VBM: voxel-based morphometry
WHO-UCLA AVLT: World Health Organization- University of California-LosAngeles Auditory Verbal Learning Test
WM: white matter
